# How do teenagers sleep? Analysis of factors related to sleep disorders in a group of Polish high school students

**DOI:** 10.1186/s12887-023-04327-0

**Published:** 2023-10-02

**Authors:** Dominika Tatar, Paweł Dębski, Bogusława Bocian, Małgorzata Bąkowska, Joanna Będkowska, Magda Tropiejko, Patryk Główczyński, Karina Badura-Brzoza

**Affiliations:** 1 Clinical ward, Department of Psychiatry, Faculty of Medical Sciences in Zabrze, Medical University of Silesia, Ul. Pyskowicka 47, Katowice, Tarnowskie Góry 42-600 Poland; 2 Institute of Psychology, Humanitas University, Sosnowiec, Poland; 3 Department of Psychiatry, Faculty of Medical Sciences in Zabrze, Medical University of Silesia, Students’ Scientific Association, Katowice, Poland; 4 Department of Endocrinology and Metabolic Diseases, Regional Specialist Hospital No. 3, Rybnik, Poland; 5 Department of Rheumatology and Autoimmune Diseases, Rheumatology Center, Ustroń, Poland; 6 Department of Neurology, SPZOZ MSWIA, Rzeszów, Poland

**Keywords:** Insomnia, Adolescents, Factors affecting sleep

## Abstract

**Summary:**

Insomnia is the most common sleep disorder in the adolescent population. Resulting from a complex interplay of genetic, biological, social, and environmental factors, it affects disturbances in everyday functioning in many aspects of life. The aim of the study was to analyze the factors related to the occurrence of insomnia among high school students.

**Material:**

The study was conducted among 2364 high school students aged between 14 and 19 years old (the average age 17.92 ± 1.10), of which there were 2068 girls and 295 boys.

**Method:**

Athens Insomnia Scale (AIS) and authorial demographic data questionnaire involved questions about physical activity during the day, smoking, frequency of upper respiratory tract infections, problems with concentration and memory, school achievements, and the use of mobile devices at bedtime were used to assess examined parameters. The scale and questionnaires were shared on-line via social media.

**Results:**

Analyzing the results obtained in the study group, the average score of 8.42 ± 4.16 was obtained in the AIS, which allows the assessment of sleep disorders bordering on normal. After division into groups of girls and boys, 8.38 ± 4.56 points were obtained, respectively for girls and 8.43 ± 4.27pts. for boys - the difference was not statistically significant. Similarly, statistically significant differences were not found when dividing the groups into the one in which adolescents used the phone just before bedtime and the one that did not use it. The act of sleeping alone or with another person in the room also did not differentiate the groups statistically, as well as the place of residence. Statistically significant worse results in the AIS scale were obtained by people who declared worse physical activity during the day and smoking cigarettes, as well as those who reported more frequent problems with concentration and memory, had worse school performance and suffered from upper respiratory infections more often.

**Conclusions:**

1). Sleep disorders may be related to factors such as smoking cigarettes or lack of physical activity, as well as difficulties in concentrating attention, memory disorders or worse academic performance and a tendency to contract upper respiratory tract infections. 2). Elimination of factors that may adversely affect the quality of sleep is particularly important in the group of adolescents, in whom the developing structures of the central nervous system may be particularly sensitive to deficiencies in this area. 3). Exploration of the interplay of study duration, screen time, and semester-specific stressors on sleep quality could yield further insights.

## Introduction

Insomnia is the most common sleep disorder not only in the adult population but also among adolescents. According to the International Classification of Diseases 10th Revision (ICD-10), inorganic insomnia is defined as insufficient quantity and/or quality of sleep, occurring at least 3 times a week for at least one month, not caused by somatic and/or neurological causes, which results in deterioration of well-being and functioning [1, 2]. On the other hand, according to the DSM V criteria, insomnia is defined as the predominant sleep dissatisfaction despite having an appropriate opportunity to sleep, including difficulties in falling asleep and / or staying asleep and/or waking up early with an inability to return to sleep. Symptoms should persist for at least 1 month and cause significant suffering to the patient or limit his/her functioning, both socially and professionally [2, 3]. Insomnia is rarely an isolated symptom, it frequently coexists with other mental disorders such as depression, anxiety, somatization disorders or post-traumatic stress disorder (PTSD) [5, 12] and correlates with harmful usage and addiction to alcohol, caffeine, tobacco or marijuana - substances that cause activation of the CNS, hindering falling asleep and restful sleep [2]. Insomnia also often occurs in the course of somatic diseases - it accompanies chronic pain, cardiovascular diseases (hypertension, coronary artery disease), respiratory diseases (obstructive lung diseases), digestive system diseases (reflux disease) or metabolic diseases (obesity, diabetes). Applied pharmacotherapy of somatic diseases can also lead to sleep disorders [2, 5, 13, 14]. Despite the fact that these problems most often occur in the elderly, the studies conducted so far have shown that it may also affect from 4% to even 39% of adolescents. Causes may be both genetic factors (higher incidence of insomnia in children of sick parents [4]), as well as the biological, social or environmental ones [5]. Among biological factors changes like CNS development that causes shifts in the sleep architecture - slow reduction in the amount of slow-wave sleep and the start of the REM phase, prolongation of the N1 and N2 stages as well as the wakefulness interfered during sleep, shortening of N3 sleep associated with a sharp decrease in delta power, are taken into consideration [1, 4]. Genetically determined is also the so-called. “night owl” chronotype - increased activity shift towards evenings hours, resulting in a delay in falling asleep, difficulties in waking up and morning drowsiness [6–10]. Sleep disorders are also clearly related to gender. Female sex determines hormonal changes, resulting in greater neuroendocrine sensitivity, and thus stress reactivity affecting sleep [2, 5, 11]. Also, stress itself, as an environmental factor, is not without significance in reducing the amount and quality of sleep by activating the sympathetic nervous system and increasing cortisol secretion which puts the body in a state of agitation, which makes it difficult to fall asleep and also disturbs the continuity of sleep [5]. Recently, the tendency to use electronic devices at bedtime, which is frequent, especially in the group of young people, has become very important -exposure to artificial light in the evening and also emotional approach to the posted content may hinder the process of falling asleep [14].

The aim of the study was to analyze factors that may be associated with insomnia in a group of high school students.

Hypothesis for the research:

1. Variables as waking up at night, napping during the day, sleepiness during school hours, using mobile devices just before bedtime or intensity of physical activity during the day, smoking, academic performance, subjective problems with concentration, memory, frequency of upper respiratory tract infections have a negative influence on teenagers sleep quality.
2. Adolescents have difficulties with sleep quality.
3. Teenagers with higher BMI have worse sleep quality.

## Material

The study was conducted among 2364 Polish high school students aged between 14 and 19 years old (the average age 17.92 ± 1.10), of which there were 2068 girls and 295 boys. The inclusion criteria were age 14–19 and Polish nationality. The exclusion criteria were other than Polish nationality, lack of parental consent and any form of intellectual disablities.

Research was conducted from 1st of September 2022 to 31st of June 2022. All respondents agreed to participate in the project, the parents of those subjects who were minors also agreed to participate (Parental consent on a paper was collected in schools, then sent to authors).

Prior to data collection, schools were sent a written invitation to participate in the study. The study was conducted online all over Poland that agreed to participate in the project. Participants were informed that they could withdraw without consequence at any time during the study.

## Methods

The following research questionnaires were used in the study:

1. Athens Insomnia Scale (AIS) - The eight-item questionnaire evaluates sleep onset, night and early-morning waking, sleep time, sleep quality, frequency and duration of complaints, distress caused by the experience of insomnia, and interference with daily functioning. Respondents use Likert-type scales to show how severely certain sleep difficulties have affected them during the past month. Scores range from 0 (meaning that the item in question has not been a problem) to 3 (indicating more acute sleep difficulties). Achieving a score of 0–5 means normal, 6–10 borderline normal, and above 11 - sleep problems. The scale assesses the severity of insomnia using diagnostic criteria set forth by the International Classification of Diseases (ICD-10). The Cronbach’s alpha was 0.89 [15].
2. Authorial demographic data questionnaire. This questionnaire was designed to control sociodemographic variables that may be related to sleep problems. It consisted, among others, questions about gender, age, place of residence, close relationships, and such variables as waking up at night, napping during the day, sleepiness during school hours, using mobile devices just before bedtime or intensity of physical activity during the day, smoking, academic performance, subjective problems with concentration, memory, frequency of upper respiratory tract infections.
3. The questionnaire collected height and weight of respondents. BMI was calculated from the formula BMI = weight/height^2^. For children up to 18 years of age, the appropriate weight is determined on a percentile grid and is between the 3rd and 97th percentiles. In the study, BMI was not intended to indicate obesity, but rather to indicate the relationship between BMI and insomnia.

The study was prepared in the google form and conducted electronically. The forms were available on-line via social media.

### Statistical analysis

Standard statistical procedures were used in the analyses. The Kraskal-Wallis test and the Mann-Whitney U test were used to assess the significance of differences between the study groups. Spearman’s rank correlation coefficient was used to assess the relationships between the data. The significance level of p < 0.05 was assumed as statistically significant. Calculations were made in Statistica version 13.3.

## Results

### Description of the study group

The study group included 2068 girls aged 14 to 19, with an average age of 17.95 ± 1.09, while the number of boys participating in the study was 295, aged 14 to 19, with an average age of 17.78 ± 1.24. Despite attempts to indicate differences between the sexes, researchers were aware of the gender disproportion of the study group.

BMI was: for girls on average 17.95 ± 1.08, for boys: 17.77 ± 1.23. The mean score for both groups together was 17.92 ± 1.10. In the study, BMI was not intended to indicate proper weight.

1. *Athens Insomnia Scale (AIS)*
  Analyzing the results obtained in the study group, the average score of 8.42 ± 4.16 was obtained in the AIS, which allows the assessment of sleep disorders bordering on normal. After division into groups of girls and boys, 8.38 ± 4.56 points were obtained, respectively for girls and 8.43 ± 4.27pts. for boys. The difference was not statistically significant (Table 1.).

**Table 1:** Relationships between AIS score and examined factors among adolescents

Differentiating variables	AIS						Mann-Whitney U
	Mean	SD	Median	Mean	SD	Median	Z
Using the phone before bedtime	YES (n = 2252)			NO (n = 112)			-0.572
	8.397	4.109	8.000	8.875	5.046	8.000	
Sleeping alone in a room	YES (n = 2023)			NO (n = 341)			-0.190
	8.457	4.150	8.000	8.457	4.217	8.000	
Awakenings during the night	YES (n = 1302)			NO (n = 1062)			-7.920
	9.006	4.138	9.000	7.698	4.071	7.000	
Learning difficulties	YES (n = 1740)			NO (n = 624)			-18.141
	9.311	4.084	9.000	5.931	3.259	5.000	
Memory problems	YES (n = 1165)			NO (n = 1199)			-16.736
	9.842	4.048	10.000	7.037	3.783	7.000	
Worse academic performance	YES (n = 1756)			NO (n = 608)			-17.038
	9.247	4.092	9.000	6.028	3.348	5.000	
Daytime naps	YES (n = 1811)			NO (n = 553)			-5.744
	8.681	4.1569	8.000	7.562	4.052	7.000	
Smoking	YES (n = 736)			NO (n = 1628)			-7.689
	9.344	4.080	8.000	8.001	4.128	8.000	

AIS – Athens Insomnia Scale, SD – standard deviation

**Fig. 1:**
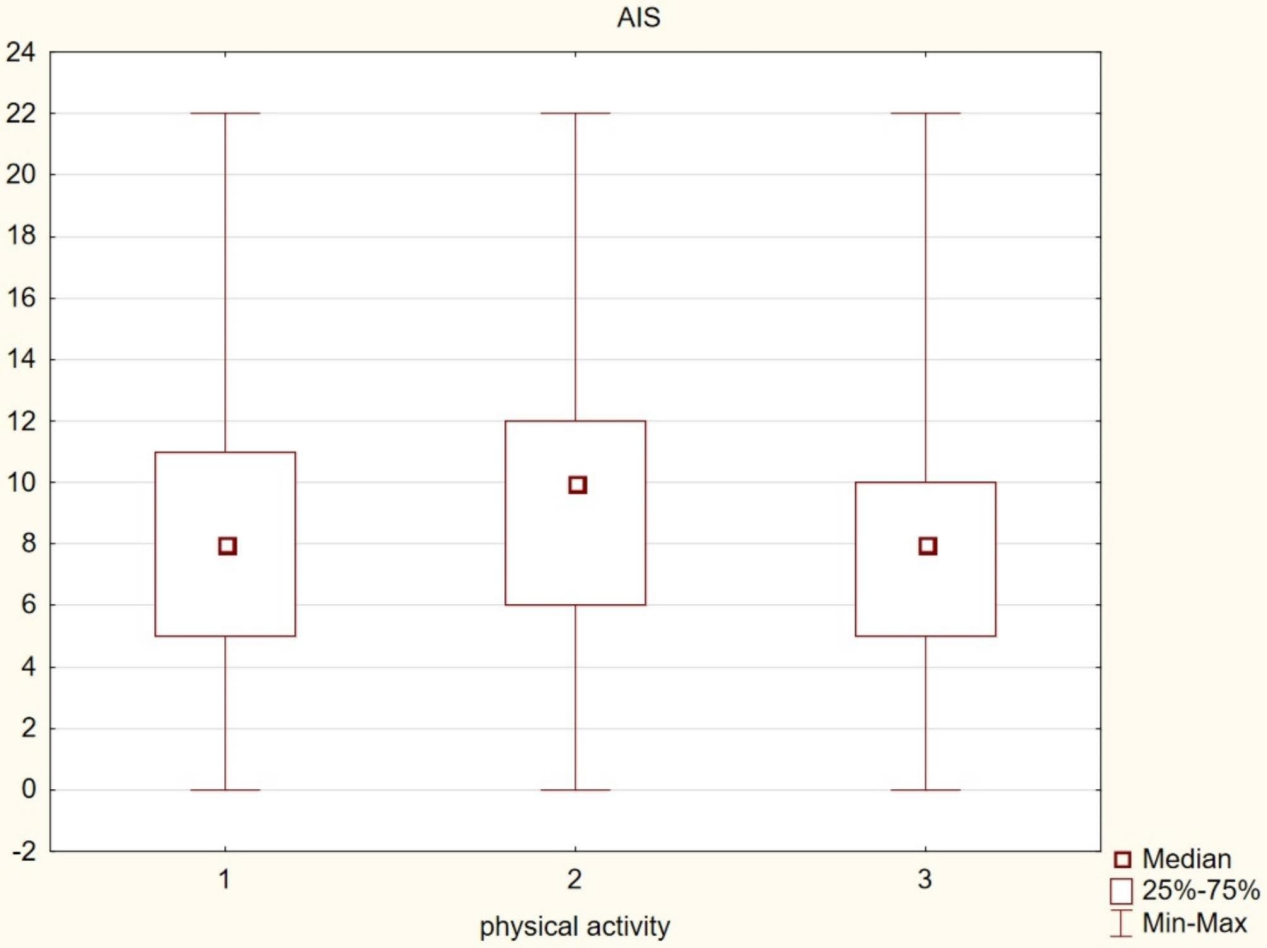
Physical activity and insomnia among adolescents. Note: 1 – physical activity within PE, 2 – lack of physical activity, 3 – physical activity as part of PE and extracurricular activities

**Fig. 2:**
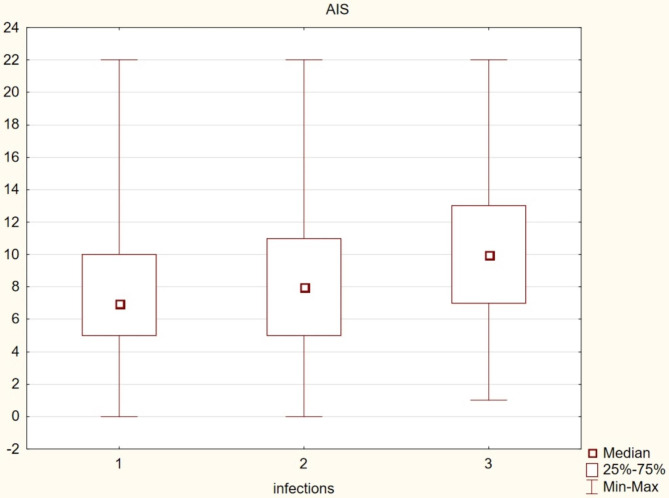
The incidence of infections, mainly of the upper respiratory tract, and insomnia among adolescents. Note: 1 –maximum once a year, 2–2–4 times a year, 3 – more than 4 times a year

2. *Analysis of the connections between AIS and BMI*
  There was no statistically significant relation between BMI and the AIS result.
3. *Analysis of differences in the AIS between groups distinguished due to sociodemographic variables and experienced symptoms.*
  Significant results with a differentiating variable of a multi-category nature were presented using Figs. (1 and 2). Statistically significant differences were not found when dividing the groups into the one in which adolescents used the phone just before bedtime and the one that did not use it. The act of sleeping alone or with another person in the room also did not differentiate the groups statistically, as well as the place of residence.
  However, statistical differences were noted in the following variables:
  frequent getting out of bed during the night (over 3 times): people who got up at night (n = 1302) presented worse results on the AIS scale (p = 0.00);
  shortening the time of effective learning during the day: people who complained about shortening the time of learning (n1707) scored worse on the AIS scale (p = 0.00);
  memory problems: people who complained about subjective memory problems (n = 1165) achieved significantly worse results on the AIS scale (p = 0.00);
  learning results: people who declared average and poor grades (n = 1756), achieved worse learning results than people who declared good and very good achievements (p = 0.00).
  difficulties in concentration hindering the learning process: people who complained of difficulties in concentration (n = 1740) showed significantly worse results on the AIS scale (p = 0.00);
  daytime naps: people who confirmed daytime naps (n = 1811) scored worse on the AIS scale (p = 0.00);
  cigarette smoking: people who declared smoking cigarettes (n = 736) obtained significantly worse results on the AIS scale (p = 0.00);
  physical activity during the day: adolescents who declared a complete lack of activity, including PE classes at school (n = 504), achieved worse results on the AIS scale compared to people who practiced a physical activity during PE classes (n = 752), (p = 0.00), as well as people who declared both types of activity; as part of PE and extracurricular physical activity (n = 1118), (p = 0.00), (Fig. 1)
  the frequency of upper respiratory tract infections: people declaring more than 4 infections per year (n = 510) achieved significantly worse results on the AIS scale than those suffering 2–4 times a year (n = 1041), (p = 0.00) and those suffering no more than once a year (n = 813) (p = 0.00), (Fig. 2).

## Discussion

Sleeping disorders are a heterogeneous group of frequently occurring health problems that are of interest to doctors of various specialties. Insomnia affects 6–15% of people, increases the risk of developing depression and suicidal behavior, cardiovascular diseases and type 2 diabetes. A special age group in which sleep problems may occur are teenage children. In a study conducted on a group of 2,364 children aged between 14 and 19, the average AIS score was 8.42 ± 4.16, which allows for the assessment of sleep disorders bordering on normal. There was no statistically significant difference between the results obtained by girls and boys. Some researchers indicate a higher incidence of insomnia in the female population. Richardson et al. showed that in women with tendencies to perfectionism, the stress experienced for this reason may contribute to the reduction of sleep [11]. Hormonal factors are another factor that predisposes the female sex to more frequent sleep disorders [5, 11, 16], although research shows that this factor may be important at a later age [16–18]. In the study group of this study, girls and boys are just after puberty, therefore hormonal activity enters the stabilization phase and may play a lesser role in the occurrence of sleep disorders [19]. Similarly, BMI was not associated with insomnia in the study group, although such a relation is often emphasized in scientific studies, but always in the context of the negative impact of overweight or obesity on the quality of sleep [20–22]. In this study, the BMI of the subjects was within the normal range for age, hence the probable lack of correlation with the AIS score. Another factor affecting the quality of sleep is the use of psychoactive substances. Young people who declared smoking had worse results on the AIS scale. Kasperczyk et al. also mention coffee drinking, regular alcohol consumption and the use of other stimulants as significant factors affecting the poorer quantity and quality of sleep [23]. So far, the relation between the amount and quality of sleep and physical activity remains unknown. The study showed that adolescents who declared a complete lack of activity, including physical education classes at school, achieved worse results on the AIS scale compared to people who showed physical activity. Similar conclusions were drawn by Stone and Gomes et al. in their research. Stone pointed out that children sleeping less than 9 h a day undertook a smaller amount of lighter activity compared to peers sleeping the recommended number of hours. In turn, Gomes et al. observed that people who sleep longer lead a more active lifestyle. It has also been proven that regular physical activity itself improves the quantity and quality of sleep, while neglecting it in favor of using electronic devices contributes to the occurrence of insomnia [24, 25]. It is puzzling that the conducted study did not show a connection between the use of electronic devices (including the Internet) and insomnia. Many researchers pay attention to such a relationship. Woods and Scott found that the two main sleep deprivation factors for teenagers using social media platforms were bedtime use and emotional attachment to content. They also drew attention to the relation between the amount of time spent using the media and the severity of sleep disorders [14]. Similar conclusions were drawn by de Zambotti M et al. [5]. They also put attention on the consequences, which include, among others, “social jet lag” as a common phenomenon among young people, where they “make up for sleep” on weekends. Many authors pay attention to the consequences of insomnia, especially the impact on the everyday functioning of adolescents. In a study conducted in Finland in the years between 1984 and 2011, including over a million questionnaires (Kornholm et al.), it was found that insomnia and its accompanying symptoms significantly affect the deterioration of school performance. A similar thesis was put forward by Li et al., who conducted a cohort study on a group of 612 students, in which it was found that insufficient amount and quality of sleep causes deterioration of attention concentration, cognitive abilities and motivation to learn, and as a result, achievement of worse results in school, which was particularly noticeable in the pediatric population, who reported sleeping less than 9 h. Similar conclusions were drawn by Argentinian researchers (Perez-Lloret et al.), who pointed out that secondary drowsiness during the day significantly reduces the concentration of attention. In a study conducted among 4966 adolescents in Shanghai, it was shown that difficulties with falling asleep caused difficulties with focusing attention, but also the occurrence of frustration and poorer quality of relationships with peers. On the other hand, difficulties with maintaining sleep, apart from the deterioration of attention, caused less interest and weaker motivation to learn [26–30]. It also seems important that in this study, people declaring more than 4 infections per year achieved significantly worse results on the AIS scale compared to people suffering 2–4 times a year and people getting sick no more than once a year. In his study, Kasprzak pointed to the lowering of the body’s immunity as a consequence of insomnia [31]. In other studies (Wichniak et al.) it was also pointed out that insomnia, especially short-term insomnia (lasting 2–3 weeks), may be the result of an infectious disease [32]. Sleep is a physiological need, the fulfillment of which determines the proper course of mental processes, and deprivation of this need has huge biological effects on the body and affects the functioning of the individual. Therefore, it is important to care for the right amount and quality of sleep, especially in the group of adolescents, where its deficiency can have a significant impact on the developing body and mental functioning.

### Study limitations

The presented study, like any study in which the results were collected using the Internet, has certain limitations. First of all, the collection of data via online forms makes it impossible to control the course of filling out the questionnaires. Sociodemographic data questionnaires containing questions about variables that may be related to sleep disorders were based on the subjective assessment of the respondents. The limitation of the work is also the collection of research material in the field of high schools only, without creating comparison groups from other teaching units in the same age group.

The study group is unequal considering the group of boys and girls. Girls predominate in the study. For this reason, the research results refer mainly to the group of young girls, and in relation to boys they should be treated with caution. Moreover, it should be mentioned that the gathered group is not representative of the group of teenagers in Poland. The study group was collected using the convenient sampling method, which gave the researchers limited control over filling out the questionnaires by the respondents and the structure of the gathered group of teenagers.

The research was conducted in 2022, the results was not collected in specific time in semesters. The time of screen time before sleep was also not collected. Authors believes, that data could futher insights.

## Conclusions

1. Sleep disorders may be related to factors such as smoking cigarettes or lack of physical activity, as well as difficulties in concentrating attention, memory disorders or worse academic performance and a tendency to contract upper respiratory tract infections.
2. Elimination of factors that may adversely affect the quality of sleep is particularly important in the group of adolescents, in whom the developing structures of the central nervous system may be particularly sensitive to deficiencies in this area.
3. Taking the complexity of sleep patterns and their determinants into consideration - Exploration of the interplay of study duration, screen time, and semester-specific stressors on sleep quality could possibly yield further insights.

## Data Availability

The datasets generated and/or analysed during the current study are not publicly available due privacy and data protection reasons but are available from Dominika Tatar on reasonable request.
